# Effects of Antiacid Therapy on Granuloma after Transoral Type IV-VI Cordectomy in Patients with Early-Stage Glottic Cancer

**DOI:** 10.1155/2020/4178376

**Published:** 2020-08-10

**Authors:** Xiaofeng Jin, Yanyan Niu, Wei Gu, Jian Wang

**Affiliations:** ^1^Department of Otolaryngology, Peking Union Medical College Hospital, Chinese Academy of Medical Science and Peking Union Medical College, Beijing 100005, China; ^2^Department of Otolaryngology, The First Affiliated Hospital of Xinjiang Medical University, Urumchi, 830011 Xinjiang Uygur Autonomous Region, China

## Abstract

**Objective:**

To investigate whether preventive administration of a proton pump inhibitor (PPI) can reduce the occurrence and development of traumatic granuloma (TG) following type IV-VI cordectomy.

**Methods:**

We retrospectively analyzed the status of postoperative granulomas in 37 patients who underwent type IV-VI cordectomy due to glottic cancer and determined whether postoperative administration of a PPI had any impact on granuloma formation and development.

**Results:**

The percentage and number of patients with granuloma in the PPI treatment group (experimental group) at the 1st, 2nd, 3rd, and 6th month following surgery were 81.25% (13/16), 25.00% (4/16), 18.75% (3/16), and 0.00% (0/16), respectively. The percentage and number of patients with granuloma in the no-PPI group (control group) were 95.24% (20/21), 71.43% (15/21), 52.38% (11/21), and 14.29% (3/21), respectively. The granuloma percentage of the PPI treatment group was lower than that of the control group at all postoperative time points assessed. The differences were not statistically significant at the 1st month (*p* = 0.175) but were statistically significant at the 2nd and 3rd months after surgery (*p* = 0.005, *p* = 0.037).

**Conclusion:**

Preventive use of a PPI in patients after type IV-VI cordectomy can shorten the TG recovery duration and may reduce the severity of TG, but it cannot prevent TG from occurring. Our results should be confirmed by prospective randomized controlled trials with large sample sizes.

## 1. Introduction

Laryngeal squamous cell carcinoma (LSCC) is a common head and neck cancer. It had an incidence of approximately 2.04/100,000 in China between 2003 and 2007 [1], and 177,422 new cases were reported worldwide in 2018 [2]. It is estimated that there will be 245,000 new cases worldwide in 2030 [3]. Approximately two-thirds of LSCCs originate in the glottic area. The presence of hoarseness in patients with early-stage glottic cancer (GC) prompts the patients to seek medical treatment. Anatomically, the larynx is surrounded by cartilage and has sparse lymphatic tissue. As a result, patients with GC are mostly diagnosed at an early stage, which is clinically defined as T_1/2_N_0_M_0_ [4].

In recent years, open surgery has been gradually abandoned in the treatment of early-stage GC and has been replaced with transoral microsurgery (TM) or radiotherapy. TM has the advantages of being minimally invasive and having a high laryngeal preservation rate and high cost-effectiveness. Transoral laser microsurgery (TLM) is the most common surgical method used for GC, although a few studies in the literature have adopted transoral coblation microsurgery (TCM). Although TM methods differ, thermal instruments are often needed for the surgery [5–10].

Traumatic granuloma (TG) is a common complication of TM, especially after type IV-V cordectomy. Mild TG may only manifest as hoarseness, foreign body sensation, and frequent throat clearing. The severe granuloma may cause or be complicated by perichondritis, which leads to severe symptoms, such as dyspnea. Additionally, this granulation is believed to be an important factor in the formation of glottic web and larynx stenosis [11–15]. Reflux is an important factor that affects granuloma formation. Proton pump inhibitors (PPI) are often used empirically in the treatment of patients with postoperative granulomas [8, 12, 15].

We found in our previous clinical practice that patients with a wide range of resections (such as type IV-VI cordectomies) have a higher risk of postoperative granuloma. This can sometimes be very severe, even requiring temporary tracheotomy to alleviate dyspnea, which greatly and adversely impacts patients' quality of life. PPIs have been empirically used for the treatment of patients with postoperative granuloma, and evidence of its effectiveness has been published [15]. However, there has not been any research on the prevention of postoperative granuloma formation with PPIs. Therefore, we initiated PPI treatment for patients who had undergone type IV-VI cordectomies and compared the results with those of patients who had also undergone this type of surgery but were not treated with PPIs to evaluate the effect of PPI treatment for preventing postoperative granuloma formation in this patient population.

## 2. Materials and Methods

### 2.1. Patients

This was a retrospective study. Patient inclusion criteria: (i) patients with vocal cord cancer who agreed to undergo type IV, V, and VI cordectomies; (ii) patients who did not undergo preoperative and postoperative radiotherapy. Exclusion criteria: (i) patients who were complicated with diabetes and were not treated routinely; (ii) patients who used PPIs regularly; (iii) patients who continued to smoke and drink after surgery.

A total of 21 patients between January 2015 and December 2017 were recruited as the control group, who did not use PPIs immediately after surgery and did not take PPIs persistently.

A total of 16 patients between January 2017 and June 2019 were recruited as the experimental group (PPI treatment group), who took PPIs immediately after the surgery and routinely. Since this was a retrospective study, only the patients in the experimental group underwent preoperative reflux symptom index (RSI) and reflux finding score (RFS) assessments. Patients with an RSI score > 13 points and/or an RFS ≥ 7 points were considered to have laryngopharyngeal reflux disease (LPRD) [16, 17].

All patients signed an informed consent form prior to the surgery. All clinical experiments conformed to the guidelines issued by the committee on clinical research of Peking Union Medical College Hospital (PUMCH). Ethics Committee approval was obtained at PUMCH, and all patients provided specific written informed consent.

### 2.2. Surgical Procedure

According to the European Laryngological Society classification, endoscopic cordectomies include the following types: type IV (total cordectomy), type Va (extended cordectomy encompassing the contralateral cord), type Vb (extended cordectomy encompassing the arytenoid), type Vc (extended cordectomy encompassing the ventricular band), type Vd (extended cordectomies encompassing the subglottis), and type VI (extended cordectomies encompassing the anterior commissure) [11, 18]. During the surgery, the larynx was fully exposed with a self-retaining laryngoscope (Karl Storz, Tuttlingen, Germany), and the tumor was resected en bloc under direct visualization of a 12° or 30° laryngoscope (Karl Storz, Tuttlingen, Germany) using a model 7070 coblator (ArthroCare Corp, Sunnyvale, CA) with a coblation level of 7-9 and a coagulation level of 3-5.

### 2.3. Postoperative Treatment Procedure and Follow-Up

The patients in the experimental group were treated with intravenous omeprazole 40 mg per day before the recovery of oral feeding. 12 cases recovered oral feeding on the first day after surgery, and 2 cases recovered oral feeding on the third day. Then, they were given 20 mg oral omeprazole twice daily 30 minutes before breakfast and dinner for 12 consecutive weeks. The patients in the control group were not treated with PPIs, but if severe granulomas formed during follow-up and required intervention, they were also given PPIs routinely. 20 (95%) patients in the control group developed granulomas. But only 3 patients developed severe granulomas on the 2nd, 3rd, and 3rd month after surgery, respectively, and began to be given PPIs for 12 weeks, the same as the experimental group, while the other 18 cases in the control group were not given PPIs throughout the whole follow-up period. All patients were treated with cefuroxime at 250 mg twice daily for 1 week to prevent infection. After the surgery, the patients were required to quit smoking and drinking and engage in reasonable vocal use.

The follow-up procedure included regular checkups at 1, 2, 3, and 6 months after surgery. If a granuloma was detected during the 3-month checkup, the patients were followed monthly until the granuloma disappeared. Postoperative granuloma was defined as relatively smooth tissue in the surgery region that protruded from the surrounding area that may be attached to a pseudomembrane. The granuloma and the surrounding area did not have obvious vascular hyperplasia. The proportion of patients with postoperative granuloma was used as an observation indicator. Additionally, the number of unscheduled visits and the rate of reoperation, including tracheotomy and granulectomy, were recorded as indicators of granuloma severity.

During follow-up, if the granuloma was found to severely impact vocalization and/or breathing or if patients were suspected of having a recurrent tumor, the granuloma was surgically resected, and the specimen was sent for examination.

### 2.4. Statistical Analysis

Data were statistically analyzed with SPSS 17.0 software (SPSS, Chicago, IL). Nonnormally distributed quantitative data are represented as median and interquartile range and were subjected to the Wilcoxon rank-sum test. Normally distributed measurement data are represented as mean ± standard deviation and were subjected to the independent sample *t* test. Count data were subjected to the chi-squared test. Differences with *p* < 0.05 were considered statistically significant.

## 3. Results

### 3.1. Baseline Characteristics

The baseline data of the patients in the PPI treatment group and the control group are shown in Table 1. Among the 16 patients in the PPI treatment group, 15 patients were male and 1 patient was female, with an average age of 62.44 ± 5.9 years; 12 patients were at the T_1_ stage, and 4 patients were at the T_2_ stage; 9 patients underwent type IV surgery, 3 patients underwent type V surgery, and 4 patients underwent type VI surgery. Among the 21 patients in the control group, 20 patients were male, and 1 patient was female, with an average age of 63.86 ± 5.1 years; 16 patients were at the T_1_ stage, and 5 patients were at the T_2_ stage; 12 patients underwent type IV surgery, 6 patients underwent type V surgery, and 3 patients underwent type VI surgery. The two groups of patients did not differ significantly in the baseline conditions (Table 1).

### 3.2. Postoperative Granulation

**Figure 1:**
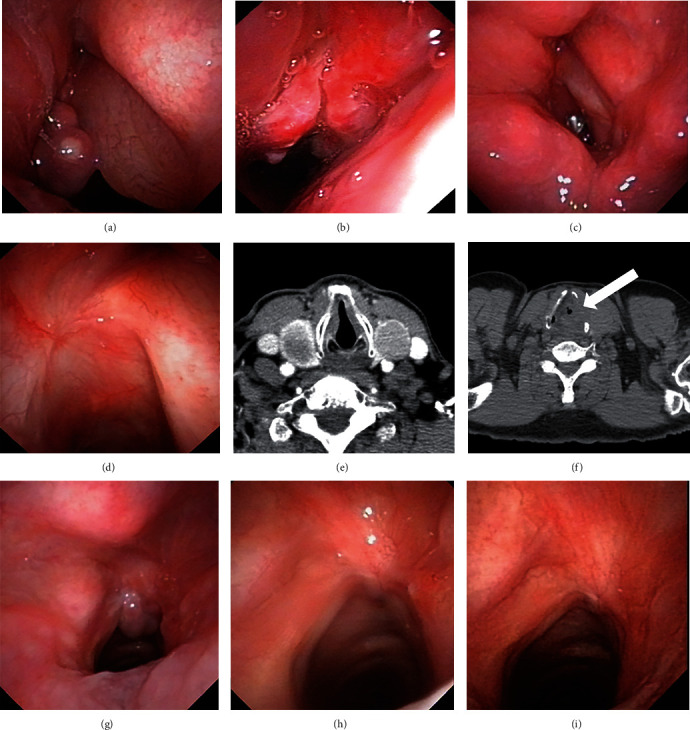
(a–f) Shows a typical case in the control group, who was a male patient for 64 years old, with glottic cancer T2N0M0 at the left side, followed by type V cordectomy. (a) One month after surgery, a granuloma was found in the left vocal cord under a fibrolaryngoscope. (b) 3 months after surgery, the granuloma enlarged and the right vocal cord become edema obviously. (c) 6 months after surgery. (d) 24 months after surgery. (e) Computerized tomography (CT) scan taken before surgery. (f) 3 months after surgery, chondronecrosis was found in the CT scan, where the white arrow points out. (g–i) Shows a typical case in the experimental group, who was a male patient for 56 years old, with glottic cancer T2N0M0 at the right side, followed by type V cordectomy. (g), (h), and (i) were taken under a fibrolaryngoscope in 1 month, 3 months, and 6 months after surgery, respectively. A granuloma could be found in (g) but disappeared in (h) and (i).

The numbers and percentage of patients with granuloma in the PPI treatment group at the 1st-, 2nd-, 3rd-, and 6th-month follow-up were 13 (81.25%), 4 (25.00%), 3 (18.75%), and 0 (0.00%), respectively. The numbers and percentage of patients with granuloma in the control group at those time points were 20 (95.24%), 15 (71.43%), 11 (52.38%), and 3 (14.29%), respectively. Although the percentage of granuloma in the PPI treatment group was lower than that of the control group at each stage, only the differences at the 2nd and 3rd months after surgery were statistically significant (*p* = 0.005 and *p* = 0.037, respectively). Only one patient (6.25%) in the PPI treatment group required a second surgery due to persistent granulation; 3 patients (14.29%) in the control group underwent a second surgery, among whom 2 patients had granuloma complicated with chondronecrosis and required to tracheotomy due to dyspnea (Figure 1). However, the difference between the two groups was not statistically significant (*p* = 0.435). In the PPI treatment group, only 2 patients had two unscheduled visits. In the control group, 8 patients had 30 unscheduled visits; among them, one patient had 9 visits due to dyspnea. The difference in the number of unscheduled visits was not statistically significant (*p* = 0.156) (Table 2).

### 3.3. Effects of PPI Treatment in Patients with LPRD in the Experimental Group

Among the 16 patients in the experimental group, 7 (43.75%) were diagnosed with LPRD according to preoperative RSI and RFS scores, and 9 patients (56.25%) did not have LPRD. The numbers of LPRD+ patients with granuloma at the 1st, 2nd, 3rd, and 6th months after surgery were 6, 3, 3, and 0, respectively, and the numbers of LPRD- patients with granuloma at these time points were 7, 1, 0, and 0. While the data showed that the percentage of granuloma in the LPRD+ patients was higher than that in the LPRD- patients, only the difference at the 3rd month after surgery was statistically significant (*p* = 0.029) (Table 3).

## 4. Discussion

Transoral microsurgery (TM) for early-stage glottic cancer (GC) can achieve oncological therapeutic effects similar to those of radiotherapy. TM has the advantages of being minimally invasive and having a high laryngeal preservation rate and a low tracheotomy rate; its disadvantage includes postoperative complications such as bleeding, infection, airway burns, and granuloma formation. Therefore, currently, the treatment selected for patients with early-stage GC is determined by the disease conditions as well as patient needs [4, 19]. Postoperative traumatic granuloma (TG) is a common complication during the healing process after TLM surgery. Severe TG may cause or be complicated by chondritis or chondronecrosis, leading to severe complications, including dyspnea. With the increase in the range of cordectomy, the incidence of TG is also significantly increased. Therefore, it is necessary to investigate ways to reduce the incidence and severity of postoperative TG [8, 15]. Like TLM, the coblation-assisted endoscopic cordectomy or TCM used in our study is also based on thermal damage, whose working temperature is 40°C-70°C, much lower than laser's working temperature which is 800°C-1000°C, and as a result, its healing process and the mechanism of TG formation are also similar to those of TLM. Current literature indicates that reflux may be an important factor in the occurrence of postoperative TG [8, 13–15]. Therefore, we aimed to investigate whether antiacid therapy could reduce TG through an analysis of the effects of PPI treatment in patients who underwent type IV-VI cordectomy, which has rarely been reported.

Our study showed that the incidences of TG after transoral surgery were as high as 81.25% and 95.24% in the two groups, which were much higher than the incidences of 18.5% reported by Nerurkar and Shah (2019) and 50.5% reported by Wang et al. (2014). The reason for this discrepancy may be that the patients in our study all underwent type IV or higher surgeries. Enlarged wounds and damage to the perichondrium or cartilage may lead to increased TG and chondronecrosis. Nerurkar and Shah reported that the incidence of TG in patients who have undergone type IV TLM was 100%, and the study by Wang et al. showed that the incidences of TG in patients who have undergone type IV and type V TLM were 78.6% and 80.0%, respectively [12, 15]. Meanwhile, the incidence of chondronecrosis was not rare (1.02%), especially on whom the surgeon had to expose the thyroid cartilage during tumor resection in TLM [20]. These studies suggest that type IV or higher surgeries lead to a high likelihood of TG occurrence.

Our study showed that PPI treatment did not suppress the formation of TG at the 1st month after surgery, but the percentage of TG in the PPI treatment group gradually became lower than that of the control group, and the differences were statistically significant at the 2nd and 3rd months after surgery. At the 6th month, granulomas disappeared in both groups. Our findings suggest that although PPI treatment cannot reduce the incidence of TG, it can significantly shorten the duration of granulomas, a finding that is similar to the study by Wang et al. [12]. Additionally, we did not observe any severe cases of TG that were complicated with dyspnea in the PPI treatment group; however, TG in 2 patients in the control group caused or was complicated by chondritis or chondronecrosis, which led to severe dyspnea. There were only 2 unscheduled visits among the PPI treatment group, compared to 30 unscheduled visits in the control group. Unfortunately, the differences in the two indicators of TG severity between the two groups were not statistically significant, although we believe PPI treatment did alleviate the severity of TG. In this study, postoperative PPI was used for 12 weeks, referring to the antiacid duration 4-24 weeks in a medical routine of vocal process granulation and laryngopharyngeal reflux disease (J. R. Lechien's report in 2019 and Chinse experts consensus on diagnosis and treatment of laryngopharyngeal and reflux disease in 2015) [21]. The results showed that PPI could shorten the recovery time and potentially prevent severe complications.

Our study showed that the percentage of TG did not differ significantly between the LPRD+ and LPRD- patients (*p* = 0.833). However, it took longer for granulomas to disappear in LPRD+ patients than in LPRD- patients, and the difference in the number of patients with granulomas was statistically significant between the two groups at 3 months after surgery (*p* = 0.029). The reason for this phenomenon may be that, during the early stage of recovery after vocal cord injury, changes in the extracellular matrix are the main manifestation, and injury impacts a lot, while acid reflux has a little effect. Acid reflux may impact the repair process in the middle and late stages [22, 23].

This was a retrospective nonrandomized controlled study with a small sample size. We only analyzed granuloma formation in the patients and did not assess their oncological outcome. We based the diagnosis of LPRD on RSI and RFS scores and did not perform dual-probe 24-hour pH monitoring. Therefore, we were unable to determine whether nonacid reflux had an impact on the formation and development of postoperative granuloma.

## 5. Conclusion

The preventive use of PPI in patients who have undergone type IV-VI cordectomy cannot reduce the incidence of TG, while it can shorten the TG recovery duration and may also reduce the severity of TG. Our findings should be confirmed by prospective randomized controlled studies with larger sample sizes.

## Figures and Tables

**Table 1:** General information.

Variable	PPI (*n* = 16)	Control (*n* = 21)	*p* value
Sex (M/F)	15/1	20/1	0.685
Age (years)	62.44 ± 5.9	63.86 ± 5.1	0.441
Tumor staging (T1a/T1b/T2)	9/3/4	13/3/5	0.920
Surgery type (IV/V/VI)	8/6/2	12/6/3	0.848

**Table 2:** Percentage and severity of granuloma in the two groups.

	1st month	2nd month	3rd month	6th month	Resurgery	Number of unscheduled visits as median and interquartile range
PPI treatment group	13/16	4/16	3/16	0/16	1/16	0 (0.0)
Control group	20/21	15/21	11/21	3/21	3/21	0 (0.1)
*p* value	0.175	0.005^a^	0.037^a^	0.115	0.435	0.156

Note: ^a^indicates that the difference is statistically significant.

**Table 3:** Percentage of granuloma in patients with or without LPRD in the experimental group.

	1st month	2nd month	3rd month	6th month
LPRD+ (*n* = 7)	6	3	3	0
LPRD- (*n* = 9)	7	1	0	0
*p* value	0.833	0.146	0.029^a^	—

Note: ^a^indicates that the difference is statistically significant.

## Data Availability

Access to these anonymized data will be made available by the corresponding author (Dr. Jian Wang) upon reasonable request.
